# Non-pathogenic *Escherichia coli* Enhance Stx2a Production of *E. coli* O157:H7 Through Both *bamA*-Dependent and Independent Mechanisms

**DOI:** 10.3389/fmicb.2018.01325

**Published:** 2018-06-15

**Authors:** Lingzi Xiaoli, Hillary M. Figler, Kakolie Goswami Banerjee, Christopher S. Hayes, Edward G. Dudley

**Affiliations:** ^1^Department of Food Science, The Pennsylvania State University, University Park, PA, United States; ^2^Huck Institutes of Life Sciences, The Pennsylvania State University, University Park, PA, United States; ^3^Department of Molecular, Cellular, and Developmental Biology, University of California, Santa Barbara, Santa Barbara, CA, United States; ^4^Center for Immunology and Infectious Disease, The Pennsylvania State University, University Park, PA, United States

**Keywords:** *E. coli* O157:H7, commensal *E. coli*, Shiga toxin, Stx2a, BamA

## Abstract

Intestinal colonization by the foodborne pathogen *Escherichia coli* O157:H7 leads to serious disease symptoms, including hemolytic uremic syndrome (HUS) and hemorrhagic colitis (HC). Synthesis of one or more Shiga toxins (Stx) is essential for HUS and HC development. The genes encoding Stx, including Stx2a, are found within a lambdoid prophage integrated in the *E. coli* O157:H7 chromosome. Enhanced Stx2a expression was reported when specific non-pathogenic *E. coli* strains were co-cultured with *E. coli* O157:H7, and it was hypothesized that this phenotype required the non-pathogenic *E. coli* to be sensitive to *stx*-converting phage infection. We tested this hypothesis by generating phage resistant non-pathogenic *E. coli* strains where *bamA* (an essential gene and Stx phage receptor) was replaced with an ortholog from other species. Such heterologous gene replacement abolished the ability of the laboratory strain *E. coli* C600 to enhance toxin production when co-cultured with *E. coli* O157:H7 strain PA2, which belongs to the hypervirulent clade 8. The extracellular loops of BamA (loop 4, 6, 7) were further shown to be important for infection by *stx2a-*converting phages. However, similar gene replacement in another commensal *E. coli*, designated 1.1954, revealed a *bamA*-independent mechanism for toxin amplification. Toxin enhancement by 1.1954 was not the result of phage infection through an alternative receptor (LamB or FadL), lysogen formation by *stx2a*-converting phages, or the production of a secreted molecule. Collectively, these data suggest that non-pathogenic *E. coli* can enhance toxin production through at least two mechanisms.

## Introduction

Shiga toxin-producing *Escherichia coli* (STEC) are estimated to cause more than 265,000 illnesses annually in United States, with 3,600 hospitalizations and 30 deaths (Centers for Disease Control and Prevention (CDC), 2012). The foodborne pathogen *E. coli* O157:H7 is a notorious serotype of STEC which continues to cause various multistate outbreaks. Ingestion of a low infectious dose of <100 cells (Tilden et al., 1996) leads to outcomes ranging from asymptomatic carriage, bloody diarrhea, to life-threatening renal complications of hemolytic uremic syndrome (HUS) (Rangel et al., 2005; Tarr et al., 2005). Cattle are the natural reservoir of *E. coli* O157:H7 and asymptomatic carriers (Borczyk et al., 1987). Accordingly, beef is the primary food linked to outbreaks, however, *E. coli* O157:H7's persistence in water, soil, and manure enhances its transmission to food such as fresh produce (Hilborn et al., 1999).

Shiga toxin (Stx) is required for progression of disease to severe outcomes including HUS. It is an AB_5_ toxin, whose B pentamer binds to globotriaosylceramide (Gb3) on host cell membranes (Waddell et al., 1988). The enzymatic A subunit is delivered into the cytoplasm of eukaryotic cells, and inhibits protein synthesis, resulting in apoptosis and cell death (Saxena et al., 1989; Sandvig and Van Deurs, 1992). Stx has two immunologically distinct isoforms, Stx1 and Stx2. They share 56% identity at the amino acid sequence level (Jackson et al., 1987), however Stx2 is 400-fold more potent than Stx1 (Tesh et al., 1993) and more likely to be associated with severe disease outcomes (Kawano et al., 2008). Seven allelic variants of Stx2, from Stx2a to Stx2g have been described (Scheutz et al., 2012). Epidemiological investigation showed that Stx2a is more frequently found in strains causing HUS (Friedrich et al., 2002; Persson et al., 2007).

The genes encoding Stx are present in temperate prophages (Hayashi et al., 2001; Perna et al., 2001). During the lytic cycle, the prophage excises from the host chromosome, utilizes the host machinery to replicate, assembles new virons, and eventually lyses the host. Conversely, in the lysogenic state, the prophage replicates along with the host without causing substantial cell lysis. The switch between cycles is controlled by *cI*. During the lysogenic state, the repressor CI dimerizes and inhibits transcription from the promoters P_L_ and P_R_. However, when the SOS response is triggered by DNA damage, activated RecA cleaves CI repressor and de-represses P_L_ and P_R_, leading to prophage induction, Stx expression and cell lysis (Waldor and Friedman, 2005).

The progeny *stx*-converting phages may infect other *E. coli* strains after adsorbing to the outer membrane proteins including BamA (Watarai et al., 1998; Smith et al., 2007). BamA is essential for outer membrane protein biogenesis (Wu et al., 2005) and exists in all members of the Enterobacteriaceae family. While the amino acid sequence of BamA is nearly invariant between strains of *E. coli*, the extracellular loops 4, 6, and 7 exhibit heterogeneity between different species (Smith et al., 2007). Genetic experiments supporting BamA as a *stx2*-converting phage receptor have been difficult to perform since it is an essential gene. However, Ruhe et al. (2013) developed an approach for deleting the chromosomal copy of *bamA* by complementing *in trans* with that from *E. coli* or other Enterobacteriaceae. This system was used to identify extracellular loops 6 and 7 of BamA as critical for cell-to-cell contact of the CdiA contact-dependent growth inhibition (CDI) system.

Enhanced Stx2 production by O157:H7 can be triggered by the addition of antibiotics. Ciprofloxacin, for instance, can increase Stx production more than 40-fold (Zhang et al., 2000). It has also been proposed that Stx production can be modulated by other members of the gut microflora (de Sablet et al., 2009; Thévenot et al., 2015). Other *E. coli* such as laboratory strain C600 were shown to produce Stx2 upon addition of *stx2a*-converting phages, leading to a model that C600 enhancement of Stx2a production requires phage infection and replication (Gamage et al., 2003). Our own previous study supported this by showing C600 increased Stx2a production of O157:H7 when the two bacteria were co-cultured, and during growth the viable cell counts for C600 decreased; this phenotype has been validated *in vitro* and *in vivo* (Goswami et al., 2015). In that study, O157:H7 strain PA2 was used as a model as it belongs to the hypervirulent clade 8 (Manning et al., 2008; Hartzell et al., 2011), and was the highest Stx2a producer of strains tested when co-cultured with C600 (Goswami et al., 2015).

Genetic evidence demonstrating that toxin amplification acts through phage infection of C600 has been lacking. Additionally, it is unknown whether this model represents the main mechanism by which commensal *E. coli* enhance Stx2a production. To further study this toxin amplification phenotype, we hypothesized that other strains of *E. coli* would amplify Stx2a production in a manner that is distinct from the one described for C600. Our objectives were: (1) to take a genetic approach to confirm that toxin amplification requires C600 to be sensitive to *stx2a*-converting phages; (2) to characterize the mechanism of Stx2a amplification by a commensal *E. coli* strain designated 1.1954, which functions through a mechanism distinct from that described for C600.

## Materials and methods

### Strains and culture conditions

All the strains and plasmids used in the study are listed in Table 1. The O157:H7 strains with “PA” designations were from the Pennsylvania Department of Health collection and were characterized previously (Hartzell et al., 2011). The commensal *E. coli* strains were obtained from the *E. coli* Reference Center (ECRC) at The Pennsylvania State University. The bacteria were routinely grown in Lysogeny-Broth (LB) broth at 37°C, and their culture stocks were kept in 10% glycerol at −80°C. The modified LB broth and modified LB agar used for co-culture experiments were additionally supplemented with 10 mM CaCl_2_. Working concentrations for antibiotics used in LB broth or agar were 100 μg/mL for ampicillin (Amp), 50 μg/mL for kanamycin (Kan), 30 μg/mL for nalixidic acid (Nal), 10 μg/mL for chloramphenicol (Cam), and 10 μg/ml for tetracycline (Tet). Spontaneous Nal resistant (Nal^R^) mutants of C600 and 1.1954 were generated by spreading centrifuged cells harvested from 10 mL of overnight cultures onto LB agar plates with Nal at 37°C for 16 h. The Nal^R^ colonies were purified by re-streaking twice on similar media.

**Table 1:** Strains, plasmids, and primers used in this study.

	**Characteristic(s)**	**Reference or source**
**BACTERIA STRAINS**		
***E. coli*** **O157:H7**
PA2	*stx2a;* clade 8	Hartzell et al., 2011
PA8	*stx2a*; clade 8	Hartzell et al., 2011
PA28	*stx2a, stx2c*; clade 8	Hartzell et al., 2011
Sakai	*stx1a, stx2a*; clade 1	Hayashi et al., 2001
EDL933	*stx1a, stx2a*; clade 3	Perna et al., 2001
**Non-pathogenic** ***E. coli***
C600	K12 derivative	Appleyard, 1954
JM109	*recA*^−^, indicator strain for plaque assay	Yanisch-Perron et al., 1985
ZK1526	microcinB17 producer	Genilloud et al., 1989
1.0328	A phylogroup; O147	ECRC
1.0342	D phylogroup; O11	ECRC
1.0322	B2 phylogroup; O6	ECRC
1.1954	B2 phylogroup; O6	ECRC
1.1968	B2 phylogroup; O21	ECRC
1.0326	D phylogroup; O77	ECRC
***bam*****A Derivatives**
C600EE	C600Δ*bam*A::*cam +*pZS21*::bamA^*E*.*coli*^*, Cam^R^ Kan^R^	This study
C600EC	C600Δ*bam*A::*cam +*pZS21*::bamA^*Enterobacter cloacae*^*, Cam^R^ Amp^R^	This study
C600ST	C600Δ*bam*A::*cam +*pZS21*::bamA^*SalmonellaTyphimurium*^*,Cam^R^ Amp^R^	This study
C600DD	C600Δ*bam*A::*cam +*pZS21*::bamA^*D*.*dadantii*^*, Cam^R^ Amp^R^	This study
D4	C600Δ*bamA::cam* +pZS21::*bamA*_Δ4_*^*E*.*coli*^*, Cam^R^ Amp^R^	This study
D6	C600Δ*bamA::cam* +pZS21::*bamA*_Δ6_ *^*E*.*coli*^*, Cam^R^ Amp^R^	This study
I4	C600Δ*bamA::cam* + pZS21::*bamA*_HA4_ *^*E*.*coli*^*, Cam^R^ Amp^R^	This study
I6	C600Δ*bamA::cam* + pZS21::*bamA*_HA6_ *^*E*.*coli*^*, Cam^R^ Amp^R^	This study
I7	C600Δ*bamA::cam* + pZS21::*bamA*_HA7_ *^*E*.*coli*^*, Cam^R^ Amp^R^	This study
C4	C600Δ*bamA::cam+* pZS21-*bamA*_Ec4_*^*E*.*cloacae*^*, Cam^R^ Amp^R^	This study
C7	C600Δ*bamA::cam+* pZS21-*bamA*_Ec6_ *^*E*.*cloacae*^*, Cam^R^ Amp^R^	This study
C8	C600Δ*bamA::cam+* pZS21-*bamA*_Ec7_ *^*E*.*cloacae*^*, Cam^R^ Amp^R^	This study
C47	C600Δ*bamA::cam+* pZS21-*bamA*_Ec4/7_ *^*E*.*cloacae*^*, Cam^R^ Amp^R^	This study
C67	C600Δ*bamA::cam+* pZS21*-bamA*_Ec6/7_ *^*E*.*cloacae*^*, Cam^R^ Amp^R^	This study
4EE	1.1954Δ*bam*A::*cam +* pZS21*::bamA^*E*.*coli*^*, Cam^R^ Kan^R^	This study
4EC	1.1954Δ*bam*A::*cam+*pZS21*::bamA^*S*.^*^Typhimurium^, Cam^R^ Amp^R^	This study
***fadL*** **Derivatives**
4F	1.1954Δ*fadL*, Nal^R^	This study
4FEE	1.1954Δ*fadLΔbam*A::*cam +* pZS21*::bamA^*E*.*coli*^*, Nal^R^ Cam^R^ Kan^R^	This study
4FST	1.1954Δ*fadLΔbam*A::*cam+*pZS21*::bamA^*S*.^*^Typhimurium^, Nal^R^Cam^R^Amp^R^	This study
***lamB*** **Derivatives**
4L	1.1954Δ*lamB*, Nal^R^	This study
4LEE	1.1954Δ*lamBΔbam*A::*cam +* pZS21*::bamA^*E*.*coli*^*, Nal^R^ Cam^R^ Kan^R^	This study
4LST	1.1954Δ*lamBΔbam*A::*cam+*pZS21*::bamA^*S*.^*^Typhimurium^, Nal^R^Cam^R^Amp^R^	This study
***stx2*** **Derivatives**
PA2T	PA2Δ*stx2::tet*, Tet^R^	This study
PA8T	PA8Δ*stx2::tet*, Tet^R^	This study
PA28T	PA28Δ*stx2::tet*, Tet^R^	This study
EDL933T	EDL933Δ*stx2::tet*, Tet^R^	This study
SakaiT	SakaiΔ*stx2::tet*, Tet^R^	This study
**PLASMIDS**		
pZS21*::bamA^*E*.*coli*^*	pZS21 derivative that expresses *E. coli bamA*, Kan^R^	Ruhe et al., 2013
pZS21*::bamA^*E*.*cloacae*^*	Expresses bamA from *Enterobacter cloacae* ATCC 13047 (*bamA ^*E*.*cloacae*^*), Amp^R^	Ruhe et al., 2013
pZS21*bamA^*S*.^*^Typhimurium^	Expresses *bamA* from *Salmonella enterica* serovar Typhimurium strain LT2 (*bamA*^LT2^), Amp^R^	Ruhe et al., 2013
pZS21*::bamA^*D*.*dadantii*^*	Expresses *bamA* from *Dickeya dadantii* 3937 (*bamA*^Dd3937^), Amp^R^	Ruhe et al., 2013
pZS21::*bamA*_Δ4_ *^*E*.*coli*^*	pZS21*amp*-*bamA*^+^ derivative that deletes residues Pro^556^ – Asn^563^ within loop 4 of BamA*^*E*.*coli*^*, Amp^R^	Ruhe et al., 2013
pZS21::*bamA*_Δ6_ *^*E*.*coli*^*	pZS21*amp*-*bamA*^+^ derivative that deletes residues Phe^675^ – Lys^701^ within loop 6 of BamA*^*E*.*coli*^*, Amp^R^	Ruhe et al., 2013
pZS21-*bamA*_HA4_ *^*E*.*coli*^*	pZS21-*bamA*^+^ derivative that introduces an HA epitope into extracellular loop 4 of BamA*^*E*.*coli*^*, Amp^R^	Ruhe et al., 2013
pZS21-*bamA*_HA6_ *^*E*.*coli*^*	pZS21*amp*-*bamA*^+^ derivative that introduces an HA epitope into extracellular loop 6 of BamA*^*E*.*coli*^*, Amp^R^	Ruhe et al., 2013
pZS21-*bamA*_HA7_*^*E*.*coli*^*	pZS21-*bamA*^+^ derivative that introduces an HA epitope into extracellular loop 7 of BamA*^*E*.*coli*^*, Kan^R^	Ruhe et al., 2013
pZS21-*bamA*_Ec4_ *^*E*.*coli*^*	Expresses chimeric *bamA^*E*.*cloacae*^* in which the coding sequence for Asp^550^-Ala^567^is replaced with Tyr^550^-Thr^567^ from *bamA^*E*.*coli*^* Amp^R^	Ruhe et al., 2013
pZS21-*bamA*_Ec6_ *^*E*.*coli*^*	Expresses chimeric *bamA^*E*.*cloacae*^* in which the coding sequence for Tyr^675^ – Ser^693^ is replaced with Phe^675^ – Lys^701^ from *bamA^*E*.*coli*^*, Amp^R^	Ruhe et al., 2013
pZS21-*bamA*_Ec7_ *^*E*.*coli*^*	Expresses chimeric *bamA^*E*.*cloacae*^* in which the coding sequence for Ala^739^ – Val^752^ is replaced with Thr^747^ – Tyr^757^ from *bamA^*E*.*coli*^*, Amp^R^	Ruhe et al., 2013
pZS21-*bamA*_Ec4/7_ *^*E*.*coli*^*	Expresses chimeric *bamA ^*E*.*cloacae*^* in which the coding sequence for Asp^550^ – Ala^567^ and Ala^739^ – Val^752^ is replaced with Tyr^550^ – Thr^567^ and Thr^747^ – Tyr^757^ from *bamA*^Ecoli^, Amp^R^	Ruhe et al., 2013
pZS21-*bamA*_Ec6/7_*^*E*.*coli*^*	Expresses chimeric *bamA ^*E*.*cloacae*^* in which the coding sequence for Tyr^675^ – Ser^693^ and Ala^739^ – Val^752^ is replaced with Phe^675^ – Lys^701^ and Thr^747^ – Tyr^757^ from *bamA*^Ecoli^, Amp^R^	Ruhe et al., 2013
**PRIMERS**		
BamA-cam-For	aatgatttctctcggttatgagagttagttaggaagaacgcataataacgatggcg GTGTAGGCTGGAGCTGCTTC	This study
BamA-cam-Rev	attgatcgcctaaagtcatcgctacactaccactacattcctttgtggagaacactta ATGGGAATTAGCCATGGTCC	This study
Stx2-tet-For	atctgcgccgggtctggtgctgattacttcagccaaaaggaacacctgtat CATGTTTGACAGCTTATCATCG	This study
Stx2-tet-Rev	ttgtgacacagattacacttgttacccacataccacgaatcaggttatgcc TTTGCGCATTCACAGTTCTC	This study
Stx2-VF	cattagctcatcgggacaga	This study
Stx2-VR	gccttggtatatgcctaatctct	This study
FadL-UF	TTTTTTtctagaCCAGTTGTTCAATCACTTCAGC	This study
FadL-UR	GTAGTTAAAGTTAGTAAACAGGGTTTTCTGGCTCAT	This study
FadL-DF	CAGAAAACCCTGTTTACTAACTTTAACTACGCGTTCTGA	This study
FadL-DR	TTTTTTtctagaGCGTTTGCCTTTTTCTGTTT	This study
FadL-VF	TGCAGTCGGAGTTGTCCATA	This study
FadL-VR	CGCTTGGTCATTATGGTGTG	This study
LamB-UF	AAAAAAtctagaGGGCTTGAGACGATCACC	This study
LamB-UR	CCAGATTTCCATCTGTTTGCGCAGAGTAATCATCAT	This study
LamB-DF	ATTACTCTGCGCAAACAGATGGAAATCTGGTGGTAA	This study
LamB-DR	AAAAAAtctagaCGTGTTGCCTACCGTAACC	This study
LamB-VF	GCAATCGATCAAGTGCAGGT	This study
LamB-VR	ACATCGGCAAGACTGATTCC	This study

*ECRC, Penn State E. coli Reference Center; Amp^R^, ampicillin resistant; Cam^R^, chloramphenicol resistant; Kan^R^, kanamycin resistant; Tet^R^, tetracycline resistant; Stx2a: Shiga toxin 2a; Stx2c: Shiga toxin 2c; The species source for bamA in mutants is represented as superscript and the modification within bamA loop is represented as subscript. Mutants with bamA from E. cloacae, S. Typhimurium or D. dadantii were designated with “EC”, “ST,” or “DD”, respectively. The loop variants which had in-frame deletions are named with “D”; ones having insertions are named with “I”; chimeric bamA^E.cloacae^ with individual loop replaced by the corresponding one from bamA^E.coli^ is named with “C”. The lower case letter in primers designated with “For” or “Rev” represents homologous region while upper case letter for primer used to generate antibiotic resistant cassette; the lower case letter in primers named with “UF” or “DR” stands for overlapping XbaI site*.

### Co-culture experiment

The co-culture assay was adapted from Gamage et al. (2003). Overnight cultures of PA2 or non-pathogenic *E. coli* strains were separately diluted in LB broth to an OD_600_ of 0.05. One hundred and seventy microliters of each strain (OD_600_ = 0.05) was mixed and added to modified LB broth to a final volume of 1,020 μL. The mixture was placed in a six-well plate (BD Biosciences Inc., Franklin Lakes, NJ). PA2 or non-pathogenic *E. coli* strains alone were used as controls. The six-well plates had 2 mL modified LB agar serving as the bottom base. Co-culture of C600 and O157:H7 was selected as the positive control (Gamage et al., 2003; Goswami et al., 2015). Stx2a level and cell density were determined after 16 h incubation at 37°C. Polymyxin B (PMB) was added to bacteria samples to final concentration of 6 mg/mL, and incubated at 37°C for 10 min for intracellular Stx2a release. PMB was used to ensure quantification of total Stx2a synthesized by bacteria (Shimizu et al., 2009; Laing et al., 2012; Ogura et al., 2015). After centrifuging at 8,000 × g for 2 min, the supernatants were collected for immediate usage or stored at −80°C for later Stx2a measurement. The Stx2a production was evaluated by a receptor based enzyme-linked immunosorbent assay (R-ELISA) as described below. Viable cell counts were calculated by spreading serial dilutions in phosphate buffer saline (PBS) onto Sorbitol MacConkey agar (SMAC). On this medium, non-O157:H7 and O157:H7 formed red and white colonies, respectively. Cell counts and toxin levels reported are the average from three biological replicates. The relative abundance was reported as percentage of commensal *E. coli* in the total population after co-culture, calculated by the equation:

$$\begin{array}{l} \text{Competitive index }\left(\%\right)\;=\;\frac{\left(\text{Red colonies on SMAC}\right)*100}{\left(\text{Red colonies }+\text{ White colonies on SMAC}\right)} \end{array}$$

### Stx2a quantification using R-ELISA

For each R-ELISA run, supernatants from O157:H7 strain PA24 which produces only Stx1 was used as the negative control, while the lysate from high Stx2a-produing strain O157:H7 PA11 served as the positive control (Hartzell et al., 2011). The standard curves were generated using 2-fold serially diluted PA11 lysate or pure Stx2 (BEI resources, Manassas, VA). Any A_450_ above 0.2 was considered positive. Total protein in each unknown sample was measured by the Bradford assay (VWR Life Science, Philadelphia, PA), following the manufacturer's recommended protocol. Stx2a quantities were reported as μg Stx2a/mg total protein.

The R-ELISA was performed as described previously (Goswami et al., 2015; Yin et al., 2015). Detachable 96-well polystyrene microtiter strip plates (Thermo Scientific, Waltham, MA) were coated with 2.5 μg per well of Gb3 analog, ceramide trihexoside (CTH), for Stx2a capture. The plate was stored at 4°C overnight with blocking buffer consisting of 4% bovine serum albumin (Sigma-Aldrich, St. Louis, MO) in 0.01 M PBS with 0.05% Tween20 (PBST). Samples were added in triplicate to wells and incubated for 1 h at room temperature (RT). Ten nanograms of monoclonal mouse anti-Stx2 (Santa Cruz Biotech, Santa Cruz CA) which specifically binds to the A subunit of Stx2 was added to each well and incubated at RT for 1 h. Then, 10 ng goat anti-mouse secondary antibody conjugated to horseradish peroxidase (Santa Cruz Biotech, Santa Cruz, CA) was added to each well and incubated at RT for 1 h. Detection was accomplished using the 1-Step Ultra Tetramethylbenzidine (TMB) (Thermo-Fischer, Waltham, MA), which was equilibrated to RT in a foil-wrapped tube for at least 30 min prior to use. Next, 100 μL TMB substrate was added into each well and incubated for 10 min to allow for color development. Finally, 100 μL of stop solution (2 M H_2_SO_4_) was added to each well. The reading values of A_450_ were obtained using a DU®730 spectrophotometer (Beckman Coulter, Atlanta, GA). Between each addition of reagents above, the plate was washed with PBST for five times.

### Generations of gene knockouts

To generate *bamA* mutants in C600 or 1.1954, the approach from a previous study (Ruhe et al., 2013) was followed. The species source for *bamA* is indicated in the superscript and modifications of *bamA* loops are indicated in subscripts (Table 1). Target strains were first transformed with pZS21::*bamA*^*E*.*coli*^(Kan^R^). Next, the chromosomal *bamA* was deleted through one step recombination (Datsenko and Wanner, 2000) using the primer set of BamA-cam-For/Rev (Table 1). The transformants were selected on LB agar plates supplemented with Cam and Kan. Successful inactivation of chromosomal *bamA* was verified by PCR using primers BamA-VF/VR. The resulting *E. coli* Δ*bamA*::*cam* carrying plasmid pZS21::*bamA*^*E*.*coli*^(Kan^R^) was transformed with pZS21 (Amp^R^) harboring the *bamA*^*E*.*coli*^ variants or *bamA* from other species (*Enterobacter cloacae, Salmonella* Typhimurium, *Dickeya dadantii*). Plasmid exchange was selected on LB agar supplemented with Amp. Amp^R^Kan^S^ colonies were chosen for later experiments.

The in-frame deletion of *lamB* or *fadL* in 1.1954 (Nal^R^) was accomplished by marker exchange as previously reported (Chen et al., 2013). PCRs were designed using primer pairs LamB-UF/UR and LamB-DF/DR, which amplified 1,028 bp upstream and downstream of *lamB*, respectively. The two amplicions overlap by 28 bp including an XbaI site. About 20 ng of each PCR product and primers LamB-UF/DR were used in a second round of PCR. The final PCR product was digested with restriction enzyme XbaI, cloned into the suicide vector pDS132 (Philippe et al., 2004) and transformed into *E. coli* SM10λ*pir*. Cam^R^ colonies were selected. Plasmid (pDS132::Δ*lamB*) was further transformed into *E. coli* S17λ*pir*. Conjugation was conducted between *E. coli* S17λ*pir* (pDS132::Δ*lamB*) and 1.1954 (Nal^R^) as described before (Dudley et al., 2006). Transconjugants were selected on LB plates lacking NaCl, but supplemented with Cam, Nal and 5% (w/v) sucrose. Colonies were screened for Cam sensitivity and the correct deletion was confirmed by PCR using primers of LamB-VF/VR. The O157:H7 *stx2* mutants were generated following the one step recombination method for enterohemorrhagic *E. coli* strains (Murphy and Campellone, 2003). Primers Stx2-tet-For/Rev were used to replace *stx2* with a Tet cassette. The mutants were selected on corresponding LB agar plates and verified by PCR using primers Stx2-VF/VR.

### Plaque assay

An overnight culture of PA2 was diluted to an OD_600_ of 0.05 in LB broth. Ciprofloxacin was added to a final concentration of 45 ng/ml to promote *stx2a*-converting phage induction. After 8 h, the culture was centrifuged at 4,000 × g for 10 min and the supernatant was filtered through a 0.22 μm cellulose acetate filter (VWR, Radnor, PA). Phage was precipitated by adding one fourth volume of 20% PEG-8000/2.5 M NaCl buffer followed by overnight incubation at 4°C. The lysate was centrifuged at 4,000 × g for 1 h, and serial dilutions of phage suspensions were made in SM buffer [0.1 M NaCl, 50 mM Tris-HCl (pH 7.5), 8 mM MgSO_4_, and 0.01% gelatin]. Two-hundred liters of the indicator strain C600 was added to 100 μL of phage, and further mixed with 6 mL modified LB soft agar (0.75% agar). This was poured on top of a modified LB agar petri dish, and incubated at 42°C for 16 h followed by plaque quantification.

### Lysogenization rate

C600 (Nal^R^) or 1.1954 (Nal^R^) was co-cultured with individual O157:H7 *stx2* Tet^R^ mutant. After 16 h incubation, a 10-fold diluted culture was spread on LB agar plates containing only Nal to enumerate the total number of non-pathogenic *E. coli*, or onto plates containing both Tet and Nal to select for the lysogens. The rate of lysogen formation was calculated by using the equation:

$$\begin{array}{l} \text{Lysogen rate }\left(\%\right)=\frac{\left(\text{TetR and NalR colonies }\right){\;}^{*}100}{\left(\text{NalR colonies}\right)} \end{array}$$

### Occupancy determination for phage insertion sites

Both C600 and 1.1954 were whole genome sequenced on an Illumina MiSeq (San Diego, CA, USA). The Illumina reads were *de novo* assembled using SPAdes v3.9 (Bankevich et al., 2012) into contigs to identify potential insertion sites. Previously described primer pairs (Serra-Moreno et al., 2007) were used to locate insertion sites within the assembled genomes, as well as *E. coli* MG1655 (accession no. CP027060). Visual comparison of these regions in C600 and MG1655, which are known to lack prophage at these sites, with corresponding sequences from 1.1954, was used to assess site occupancy.

### Assay for CDI

The CDI assay followed a previously described protocol (Aoki et al., 2005). Polyethylene terephthalate (PET) track-etched membrane inserts (23 mm) of 0.4 μm pore size (Falcon, Corning, NY) were placed in six-well plates to create upper and lower culture wells. Overnight cultures of PA2 and non-pathogenic *E. coli* strains were diluted to an OD_600_ of 0.05. Diluted PA2 (3.2 mL) and non-pathogenic *E. coli* (2.5 mL) were added to the bottom and top chambers, respectively. Plates were incubated at 37°C with shaking at 130 rpm for 6 h. Both top and bottom samples were 10-fold serially diluted in PBS and 100 μL aliquots were plated onto SMAC plates to ensure no cross contamination occurred. After harvesting the cells and treating them with PMB for 5 min at 37°C, samples from the bottom chamber were centrifuged at 10,000 × g for 1 min, and supernatants were stored for immediate use or at −80°C. Stx2a levels were evaluated by R-ELISA.

### Data analysis

MS Excel was used to calculate the mean, standard deviation, and standard error; Minitab 18 was used for statistical analysis and GraphPad Prism 8 was used for generating figures.

## Results

### Commensal *E. coli* increases Stx2a production of *E. coli* O157:H7 strain PA2 in co-culture

**Figure 1:**
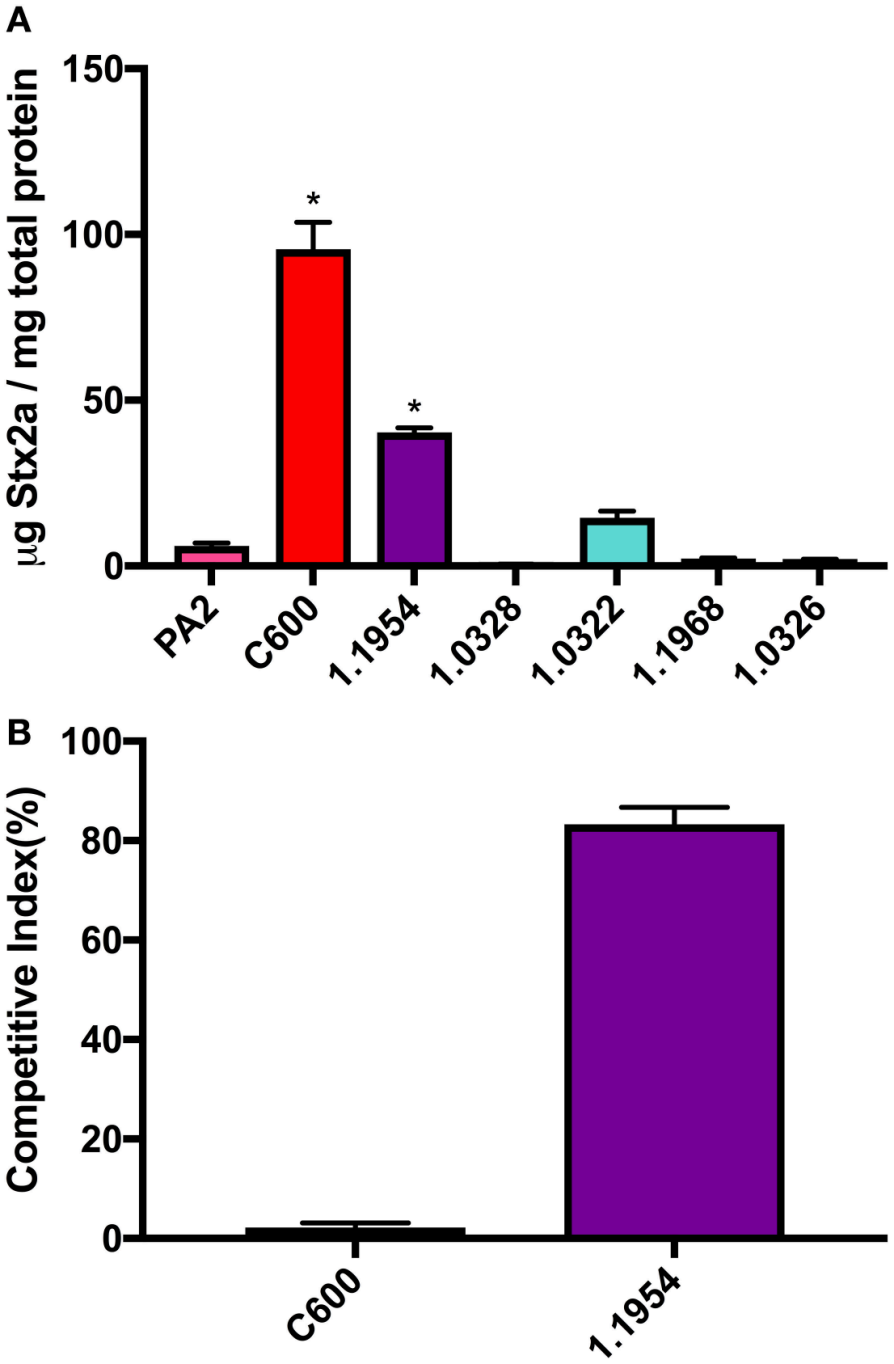
Stx2a concentration **(A)** and relative abundance of non-pathogenic *E. coli* strains **(B)** after co-culture with *E. coli* O157:H7 strain PA2. Error bars report standard error of the mean from three biological replicates. One-way ANOVA was used and the Stx2a levels in co-cultures marked with an asterisk were significantly higher than that in PA2 monoculture in pink (Dunnett's test, *p* < 0.05).

We began by testing a small collection of non-pathogenic *E. coli*, including the laboratory strain C600 and five commensal *E. coli* strains from various O types, for the ability to increase toxin production of O157:H7 strain PA2 when grown in co-culture. Co-culture of PA2+C600 produced the highest amount of Stx2a, reaching to 95.6 ± 8.1 μg Stx2a/mg total protein. Additionally, 1.1954 increased the Stx2a production in co-culture of PA2, producing 40.3 ± 1.3 μg Stx2a/mg total protein, which was significantly higher than the amount of Stx2a that PA2 produced in monoculture of 6.1 ± 0.8 μg Stx2a/mg total protein. The other four commensal *E. coli* strains, namely, 1.0322, 1.0326, 1.0328, and 1.1968 did not show significantly enhanced toxin production in co-cultures, when compared to PA2 alone (Figure 1A).

In each co-culture, both PA2 and non-pathogenic *E. coli* were inoculated at the same starting cell density. As reported previously (Goswami et al., 2015), after a 16 h co-culture, C600 abundance was 2.2 ± 0.9% of the total bacterial population, likely due to killing by the *stx2a*-converting phages produced by PA2. In contrast, an increase in cell counts of 1.1954 to 83.0 ± 2.3% was seen after co-culture with PA2 (Figure 1B). This suggested that the mechanism by which 1.1954 enhances Stx2a production by PA2 differs from that previously described for C600.

### *E. coli* C600 requires BamA for Stx2a enhancement in co-culture with PA2

**Figure 2:**
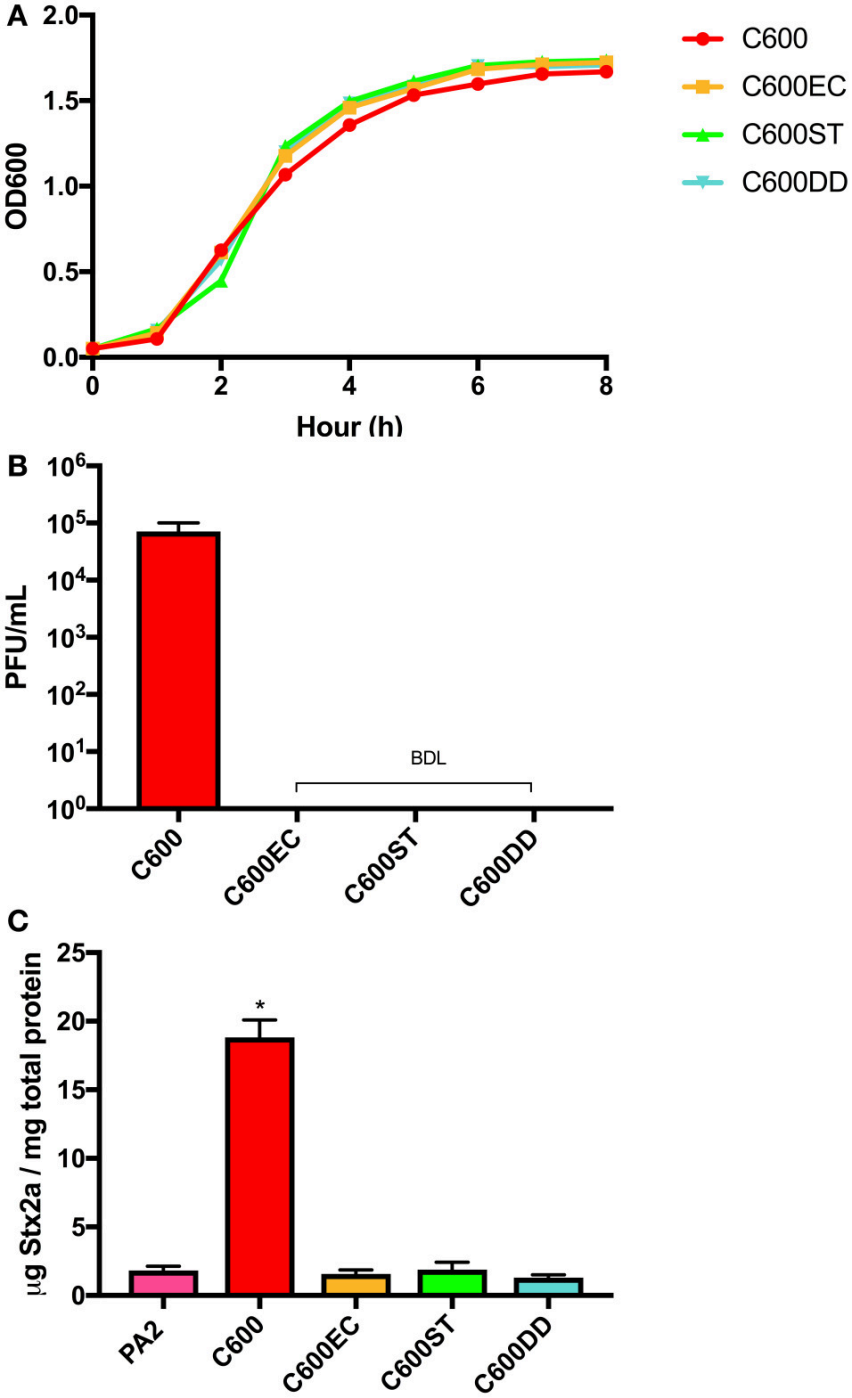
Growth curves **(A)** and plaque counts for C600 and its three *bamA* mutants **(B)** and their Stx2a levels **(C)** after co-incubating with *E. coli* O157:H7 strain PA2. C600EC, C600ST, C600DD represents three mutants expressing heterologous *bamA* from *Enterobacter cloacae, Salmonella* Typhimurium, and *Dickeya dadantii*, respectively. Error bars report standard error of the mean from three biological replicates. BDL represented below the detection limit of 10 PFU/mL. One-way ANOVA was used and Stx2a levels in co-culture marked with an asterisk were significantly higher than the PA2 monoculture (Dunnett's test, *p* < 0.05).

Several attempts to generate spontaneous phage resistant C600 derivatives were unsuccessful (data not shown), suggesting that disrupting phage adsorption requires changes to BamA beyond what can be achieved by those techniques. Using a previously published method (Ruhe et al., 2013), we generated three derivatives of C600 designated C600EC, C600ST, C600DD, in which a deletion of the chromosomal *bamA* was constructed through complementing *in trans* with plasmid-encoded *bamA* from *E. cloacae, S*. Typhimurium or *D. dadantii*, respectively. The deduced amino acid sequences of BamA from these species and *E. coli* are most divergent within the extracellular loops, which are the portions most likely involved in phage adsorption. All of these derivatives grew similarly to wild type C600 in LB broth (Figure 2A), and did not form any observable plaques when incubated with lysates containing the *stx2a*-converting phages (Figure 2B). Additionally, the concentrations of Stx2a produced during co-culture of these derivatives with PA2 were indistinguishable from that observed with PA2 alone, and significantly less than that measured in PA2+C600 (*p* < 0.05) (Figure 2C). Overall, expression of heterologous *bamA* in phage susceptible C600 rendered it resistant to phage lytic infection, providing further evidence that phage infection of C600 through BamA is required to enhance toxin production by PA2 in co-culture.

### Extracellular loops 4, 6, and 7 of BamA are required for efficient infection of *E. coli* C600 by *stx2a*-converting phages

**Figure 3:**
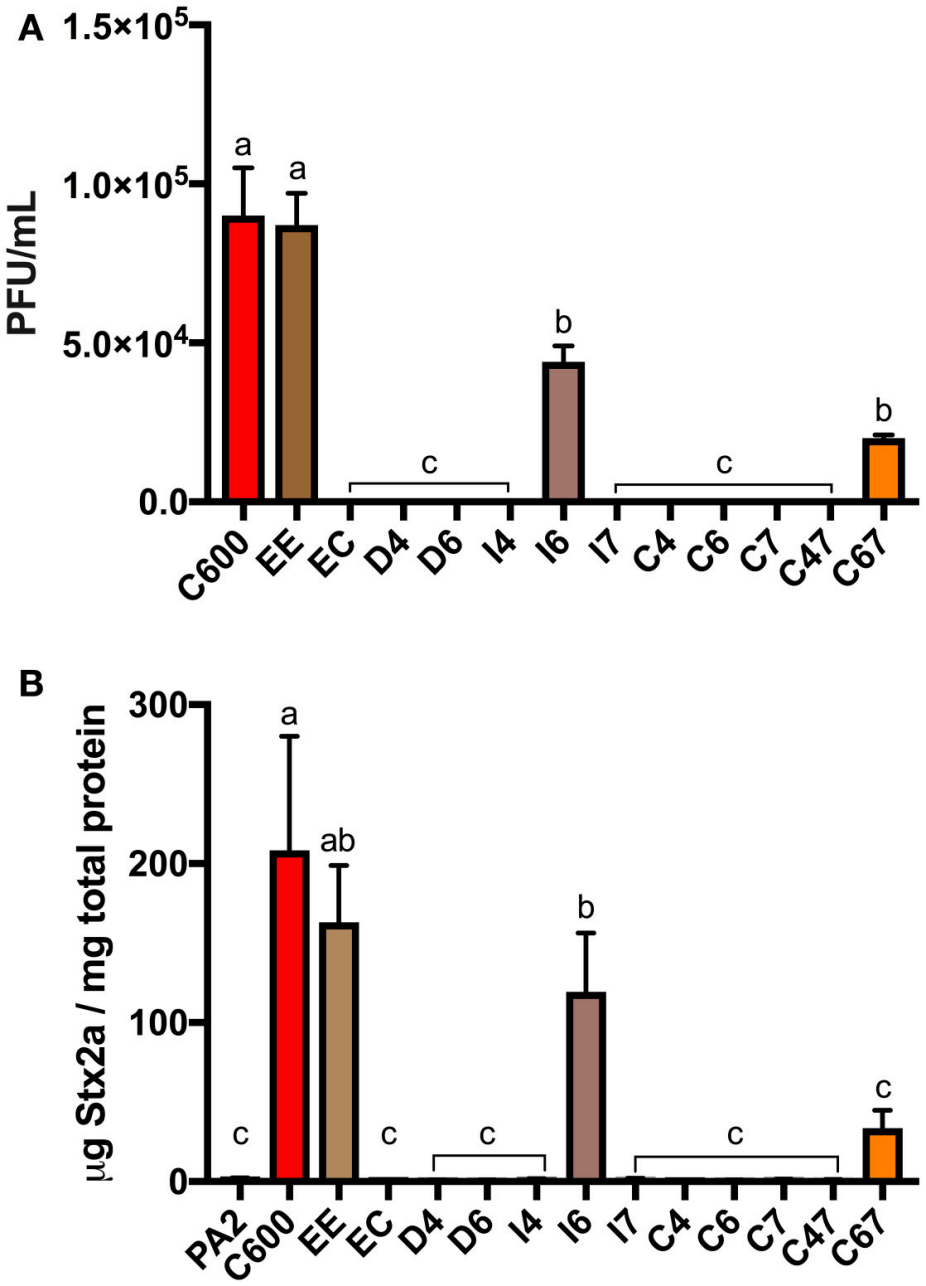
The plaque counts **(A)** for C600 and its Δ*bamA* derivatives with modified extracellular loops and Stx2a levels **(B)** after co-culture with *E. coli* O157:H7 strain PA2. Error bars were standard error of the mean from three biological replicates. One-way ANOVA was used and groups sharing the same letter (a, b, c) had no significant difference (Tukey's test, *p* < 0.05).

Using the tools developed by Ruhe et al. (2013), we could also address whether BamA loops 4, 6, and 7, which are the longest and least conserved of the extracellular loops, are needed for infection by *stx2a*-converting phages. Among the 12 variants we generated (Table 2), mutants with in-frame deletions in loop 4 or 6 (D4 and D6) as well as insertions in either loop 4 or 7 (I4 and I7) did not support the formation of detectable plaques by *stx2a*-converting phages (Figure 3A). Insertion of the HA epitope into loop 6 (I6) decreased plaque numbers by approximately 50% of that seen when C600 or EE was used as the host in the plaque assay. Strains expressing chimeric loops (C6, C7, C47) were also resistant to phage infection. The one exception was mutant C67, in which both loops 6 and 7 from *bamA*^*E*.*cloacae*^ were replaced with the corresponding sequences from *bamA*^*E*.*coli*^. This restored susceptibility to phage infection, to approximately 25% of the number of plaques seen when either C600 or EE were used as host strains.

**Table 2:** Description of loop variants of *E. coli* C600 used in this study^a^.

**Strain**	**BamA**	**Description**
EE	*bamA^*E*.*coli*^*	Entire plasmid copy of BamA*^*E*.*coli*^*
EC	*bamA^*E*.*cloacae*^*	Entire plasmid copy of BamA*^*E*.*cloacae*^*
D4	*bamA*_Δ4_*^*E*.*coli*^*	Deletion in loop 4 of BamA*^*E*.*coli*^*
D6	*bamA*_Δ6_ *^*E*.*coli*^*	Deletion in loop 6 of BamA*^*E*.*coli*^*
I4	*bamA*_HA4_*^*E*.*coli*^*	Insertion in loop 4 of BamA*^*E*.*coli*^*
I6	*bamA*_HA6_*^*E*.*coli*^*	Insertion in loop 6 of BamA*^*E*.*coli*^*
I7	*bamA*_HA7_*^*E*.*coli*^*	Insertion in loop 7 of BamA*^*E*.*coli*^*
C4	*bamA*_Ec4_*^*E*.*coli*^*	Chimeric *bamA^*E*.*cloacae*^*with loop 4 replaced by that from *bamA^*E*.*coli*^*
C6	*bamA*_Ec6_*^*E*.*coli*^*	Chimeric *bamA^*E*.*cloacae*^* with loop 6 replaced by that from *bamA^*E*.*coli*^*
C7	*bamA*_Ec7_*^*E*.*coli*^*	Chimeric *bamA^*E*.*cloacae*^* with loop 7 replaced by that from *bamA^*E*.*coli*^*
C47	*bamA*_Ec4/7_*^*E*.*coli*^*	Chimeric *bamA^*E*.*cloacae*^* with loop 4 and 7 replaced by those from *bamA^*E*.*coli*^*
C67	*bamA*_Ec6/7_*^*E*.*coli*^*	Chimeric *bamA^*E*.*cloacae*^* with loop 6 and 7 replaced by those from *bamA^*E*.*coli*^*

a *The plasmids containing above bamA alleles were previously described by Ruhe et al. (2013)*.

In accordance with these results, the co-cultures of PA2+C600 and PA2+EE produced similar levels of Stx2a, however expression of *bamA*^*E*.*cloacae*^ in place of *bamA*^*E*.*coli*^ (PA2+EC) decreased Stx2a expression to the baseline level (Figure 3B). An increase in Stx2a level was observed for PA2+C67 which had a chimeric *bamA*^*E*.*cloacae*^ with loop 6 and 7 replaced by those from *bamA*^*E*.*coli*^, but this was not significantly different from the PA2 monoculture. The PA2+I6 combination produced half of Stx2a level in PA2+C600. Together, these results suggest that the three extracellular loops (4, 6, and 7) of *bamA*^*E*.*coli*^ are essential for optimal infection of C600 by *stx2a*-converting phages.

### Commensal *E. coli* 1.1954 uses a *bamA*-independent mechanism for toxin enhancement in co-culture with PA2

**Figure 4:**
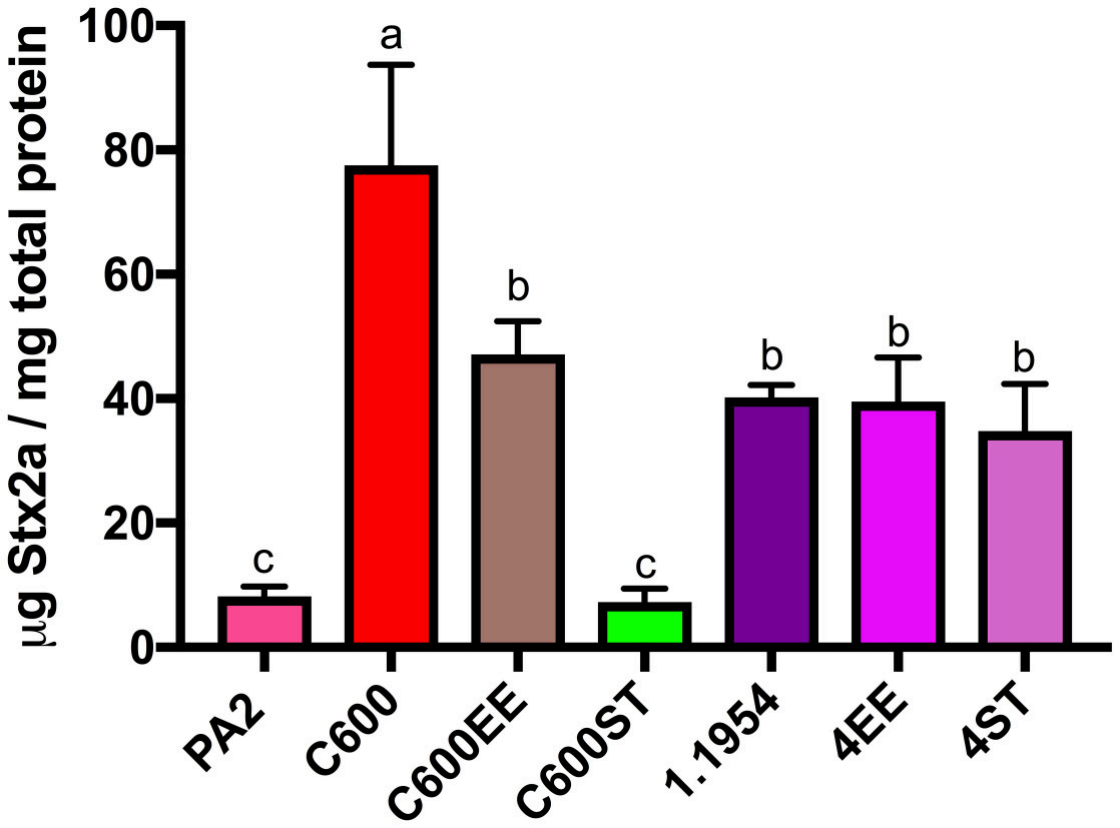
Stx2a levels after co-culture of C600 and commensal 1.1954 expressing heterologous BamA with *E. coli* O157:H7 strain PA2. ST stands for Δ*bamA* mutant with heterologous BamA from either *S*. Typhimurium and EE for Δ*bamA* mutant with homologous BamA from *E. coli*. Error bars report standard error of the mean from three biological replicates. One-way ANOVA was used and groups sharing the same letter (a, b, c) had no significant difference (Tukey's test, *p* < 0.05).

Since commensal 1.1954 was a Stx2a amplifier as well (Figure 1), we utilized the above approach to test whether *bamA* was necessary for 1.1954 mediated Stx2a amplification of PA2 in co-culture. Two *bamA* mutants were generated for 1.1954, in which the chromosomal *bamA* was deleted and complemented *in trans* by plasmid-encoded *bamA* from either *E. coli* (4EE) or *S*. Typhimurium (4ST). As expected, strains carrying *bamA*^*E*.*coli*^ (C600 or C600EE) produced significantly more Stx2a in co-cultures with PA2 than co-culture with the phage resistant strain C600ST (*p* < 0.05) (Figure 4). To the contrary, Stx2a concentrations in co-cultures of PA2+4ST and PA2+4EE were indistinguishable from that in PA2+1.1954, producing an average of 40 μg Stx2a/mg total protein (Figure 4). This suggests that commensal 1.1954 uses a *bamA-*independent mechanism for toxin enhancement in co-culture with PA2.

### Commensal *E. coli* 1.1954 is likely not infected by *stx2a*-converting phages

**Figure 5:**
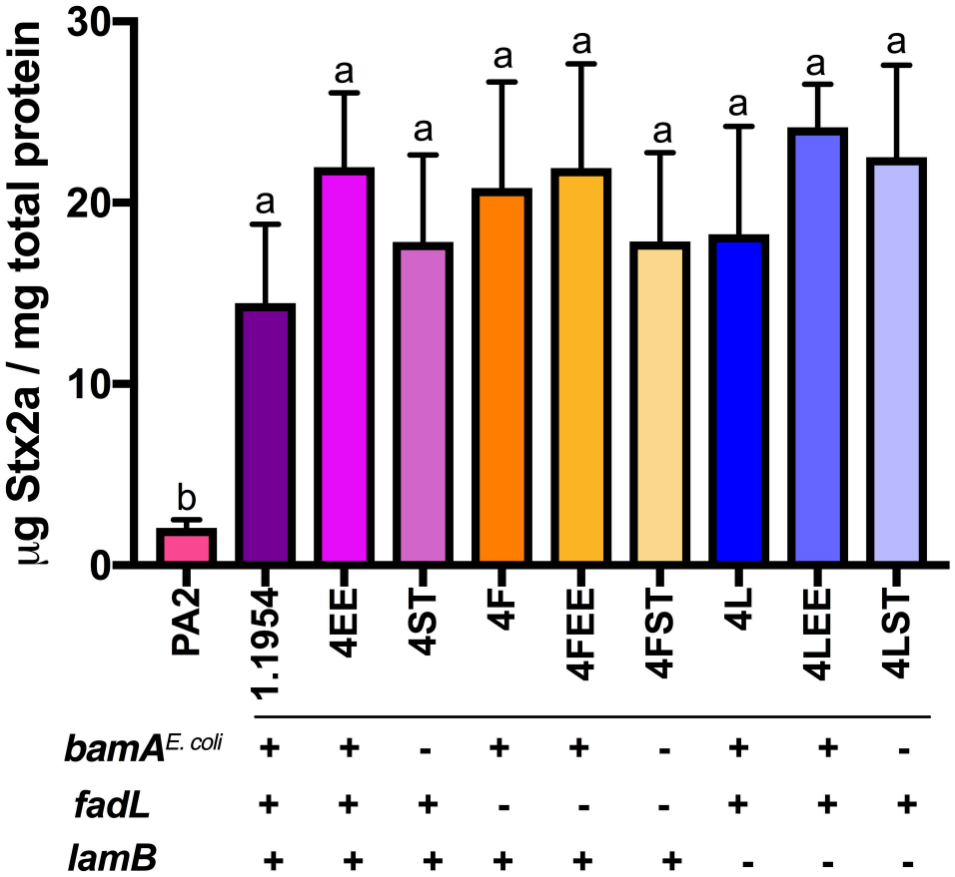
Stx2a levels in the mutants of 1.1954 after co-culture with *E. coli* O157:H7 strain PA2. In strain name, F represents Δ*fadL*, L for Δ*lamB*, EE for Δ*bamA*::*cam*+pZS21::*bamA*^*E*.*coli*^, ST for Δ*bamA*::*cam*+pZS21::*bamA*^*S*^^.Typhimurium^. The genotype is listed below the mutant names which is displayed across X axis. Error bars report standard error of the mean from three biological replicates. One-way ANOVA was used and groups sharing the same letter (a, b) had no significant difference (Tukey's test, *p* < 0.05).

Attempts to test whether 1.1954 *bamA*^*S*^^.Typhimurium^ is phage-susceptible by standard plaque assays were unsuccessful, as 1.1954 does not form a bacterial lawn when grown on the antibiotic-containing medium (data not shown). Others have suggested LamB and FadL could serve as alternative receptors for *stx*-converting phages (Watarai et al., 1998). Therefore, we generated mutants of 1.1954 with in-frame deletion in *fadL* or *lamB* (4F, 4L), or in combination with either homologous (4FEE, 4LEE) or heterologous *bamA* (4FST, 4LST). In the absence of FadL or LamB or BamA, the single knockouts (4F, 4L, 4ST) produced statistically indistinguishable levels of Stx2a when compared to PA2+1.1954 (Figure 5). Similarly, the double knockouts (4FST, 4LST) lacking BamA plus either LamB or FadL, still exhibited the toxin amplification phenotype as wild type 1.1954. These results indicate that 1.1954 does not require BamA, FadL or LamB to enhance toxin production of O157:H7.

**Figure 6:**
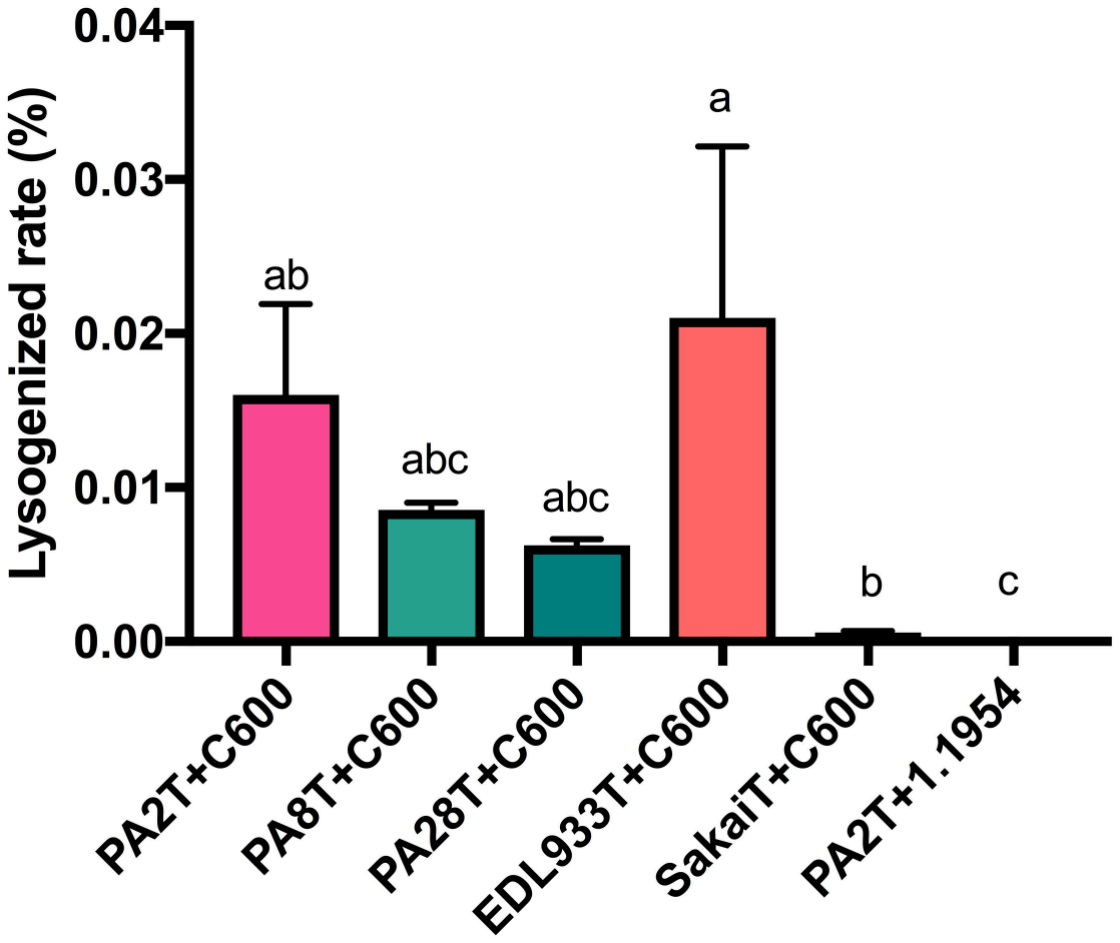
Lysogen forming rates of C600 and 1.1954 after co-culture with *stx2*::*tet* mutants of different *E. coli* O157:H7 strains. The *E. coli* O157:H7 strain was designated with the letter T indicate where a tetracycline resistant marker replaces the *stx2* gene. No lysogens were detected when any of the *E. coli* O157:H7 strains were co-cultured with *E. coli* 1.1954. Error bars report standard error of the mean from three biological replicates. One-way ANOVA was used and groups sharing the same letter (a, b, c) were not significantly different (Tukey's test, *p* < 0.05).

As an indirect measure of whether *stx2a*-converting phage infect the strain 1.1954, the PA2 mutant (PA2T) whose *stx2* was replaced with a tetracycline resistance marker was used to monitor lysogenized rates when co-culturing with either C600 or 1.1954. The average lysogen forming rate in C600 was 0.016% (Figure 6), however, no lysogen formation was observed in 1.1954. We also monitored the rate for 1.1954 at different time points during the 16 h co-culture, and no lysogens were observed at any time point (data not shown). In order to test if phage type affected lysogen formation during co-culture, several other *E. coli* O157:H7 strains carrying genetically diverse *stx2a*-converting phages (Yin et al., 2015) were also tested. In the C600 background, SakaiT had the lowest average lysogen forming rate at <0.008%, while EDL933T gave the highest of 0.021%. No difference was observed for the lysogen forming rates among PA2T, PA8T, PA28T, and EDL933T (*p* < 0.05). However, no lysogens formed in the 1.1954 background by any tested *stx2a*-converting phage (data not shown). This suggested that 1.1954 does not undergo lysogenic conversion during co-culture with O157:H7.

It was reported earlier that if the primary phage insertion site in the host strain is occupied, the *stx2*-converting phages will integrate at alternative sites (Serra-Moreno et al., 2007). Five *stx2a*-converting phage insertion sites (*sbcB, yehV, argW, yecE*, and *z2577*) were checked for occupancy in both C600 and 1.1954, the first three of which are preferred by *stx*-converting phages. Four out of five were available in 1.1954, with only *z2577* occupied. For C600, all five phage insertion sites were unoccupied. Collectively, these data suggest that *stx2a*-converting phages do not infect 1.1954.

### Commensal *E. coli* 1.1954 does not secrete DNA damaging agents that increase Stx2a of PA2

**Figure 7:**
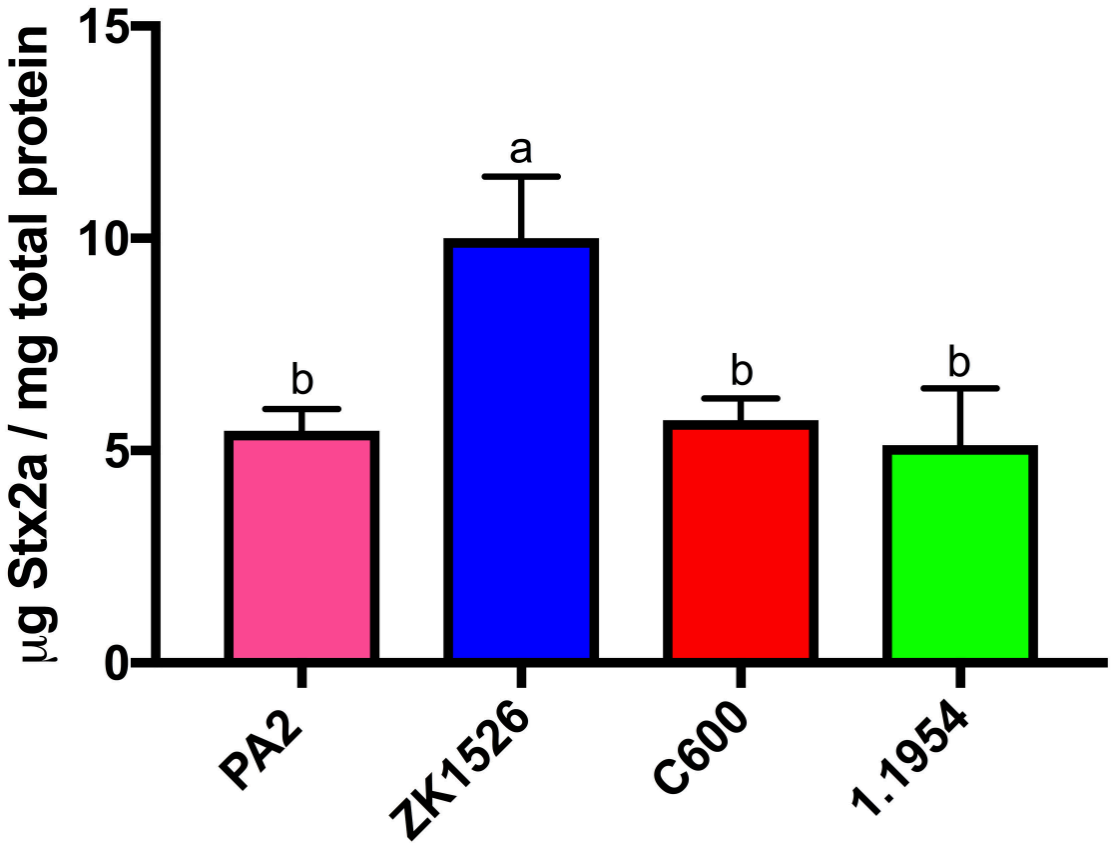
Stx2a levels of PA2 after 6 h incubation in CDI setups where PA2 grew on the bottom chamber and LB/non-pathogenic *E. coli* (ZK1526, C600, and 1.1954) on the top. Error bars report standard error of the mean from three biological replicates. One-way ANOVA was used and groups shared the same letter (a, b) had no significant difference (Tukey's test, *p* < 0.05).

To test whether secreted factors produced by 1.1954 could trigger toxin amplification of PA2, we used a modified CDI assay (Aoki et al., 2005), where non-pathogenic *E. coli* strains and PA2 were grown together while separated by a membrane. The *E. coli* strain ZK1526, which produces DNA gyrase inhibitor—microcin B17 (Genilloud et al., 1989), was selected as the positive control. As shown in Figure 7, ZK1526 promoted significantly more Stx2a production of PA2 than the negative control, PA2 alone. The toxin levels for PA2 in setups of PA2+C600 or PA2+1.1954 were as similar as the baseline level in PA2+LB. This indicated that neither C600 or 1.1954 secreted a soluble enhancer for Stx2a production of PA2.

With this result, physical contact between 1.1954 and PA2 seemed to be required for toxin amplification. Thus, we also considered a role for *cdiA*-encoding CDI systems (Aoki et al., 2005). Using BLAST, we found that 1.1954 harbors a typical *cdiBAI* operon while PA2 does not (data not shown). The deduced amino acid sequences of the carbon terminal (CT) of CdiA in 1.1954 shares 99% identity to that of uropathogenic *E. coli* (UPEC) 536, and the immunity protein CdiI shared 100% homology. Given the close relationship, we speculated the CdiA of 1.1954 is a tRNA anticodon nuclease as it is in UPEC 536 (Diner et al., 2012). If true, it seems unlikely that a tRNase is involved in increasing toxin expression (Toshima et al., 2007), and additionally this CDI system in UPEC 536 is repressed when grown in LB broth at 37°C (Aoki et al., 2010).

## Discussion

The microbiota of the human gastrointestinal (GI) tract is estimated to contain 10^14^ bacteria belonging to over 2,000 species (Thursby and Juge, 2017). *E. coli* is one species in this population, which colonizes to about 10^8^ organisms per gram of feces in healthy individuals (Tenaillon et al., 2010). Symptoms of O157:H7 infection can vary in degree of severity, and it is thought that the gut microbiota is responsible in part for modulating expression of virulence factors (de Sablet et al., 2009; Curtis et al., 2014). Commensal *E. coli* also impact toxin production of O157:H7, and a previous study reported 10% of commensal *E. coli* increased toxin produced by O157:H7 when grown in co-culture (Gamage et al., 2003). We reported previously that this phenomenon could be recapitulated *in vivo*, as mice inoculated with both O157:H7 and the non-pathogenic *E. coli* laboratory strain C600 exhibited greater signs of kidney damage and a higher mortality rate than those fed O157:H7 alone (Goswami et al., 2015). The observation that toxin production is enhanced by only a subset of commensal *E. coli*, combined with the diversity of *E. coli* strains found between individuals (Gordon et al., 2015), provides one possible explanation for individual difference in disease outcome. This study is part of a larger effort to describe commensal *E. coli* and O157:H7 interactions that alter Stx levels.

The role for phage in toxin amplification during co-culture of *E. coli* O157:H7 with other strains was previously suggested by demonstrating that addition of *stx2*-converting phages to C600 increased toxin production more than two-orders of magnitude over that seen when using a phage-resistant C600 strain (Gamage et al., 2003). Consistent with the hypothesis that toxin production requires phage to initiate a lytic infection, Goswami et al. (2015) showed that cell counts of C600 decreased upon co-culturing with *E. coli* O157:H7. Adding anti-BamA antibodies decreases phage adsorption up to 50% in a dose-dependent manner (Smith et al., 2007) and the overexpression of BamA increased the rate of lysogen formation approximately 2- to 4-fold (Islam et al., 2012), arguing that this outer membrane protein is the target for phage adsorption. Despite these data, evidence that BamA is the only receptor for *stx2a*-converging phages has been elusive, as *E. coli bamA* mutants are generally not viable (Werner and Misra, 2005). Here, we provide genetic evidence conclusively demonstrating that, at least for C600, *stx2a*-converting phages infect non-pathogenic *E. coli* exclusively through BamA. BamA from other Enterobacteriaceae share 73–93% identity to BamA^*E*.*coli*^ with the largest variation within the central region of predicted extracellular loops 4, 6, and 7 (Ruhe et al., 2013). Expression of heterologous *bamA* from *E. cloacae, S*. Typhimurium, or *D. dadantii* in place of that from C600 was sufficient to impart phage resistance, suggesting that *stx2a*-converting phage tail fibers initially bind to one or more of these loops (Figure 2). In our study, all three loops appeared to be important for phage lytic infection (Figure 3), suggesting they may come into contact with the *stx2a*-converting phage tail. In contrast, CdiA, the component of the CDI system from *E. coli* EC93 that is responsible for recognition and pore formation, recognizes target cells through BamA, in a manner that involves only extracellular loops 6 and 7 (Ruhe et al., 2013).

Although Smith et al. (2007) suggested BamA was the receptor specifically for short-tailed *stx2*-converting phage, we lack visual evidence for PA2 phage being short-tailed due to poor resolution of our transmission electron microscopy (TEM) results. Two lines of genetic evidence suggest that it is. First, Yin et al. (2015) reported that the *stx2a*-converting phage of PA2 belongs to phage type PST2-1, which is similar to phage from the German outbreak strain *E. coli* O104:H4. TEM classified the *stx2a*-convering phage of *E. coli* O104:H4 as short-tailed (Beutin et al., 2012). Secondly, Mondal et al. (2016) identified nine genes responsible for phage morphogenesis of short-tailed phage designated SP5, and these were found by BLAST to be nearly identical (>99%) on amino acid level to the homologs from phage PA2. Notably, one of these genes, ECs1228, is predicted to encode a phage tail fiber and 100% identical to the PA2 phage homolog on amino acid level.

As BamA from C600 and 1.1954 share 100% identity to one another on the amino acid level, we were surprised to find that replacement of BamA^*E*.*coli*^ with heterologous BamA^S.*Typhimurium*^ in 1.1954 did not abolish its toxin amplifying ability in co-culture with O157:H7 (Figure 4). The additional deletion of other *stx2a*-converting phage receptors (LamB or FadL) in the 1.1954Δ*bamA* background had no effects on toxin amplification as well (Figure 5). This suggests that the mechanism behind toxin amplification in 1.1954 either does not involve phage infection, or a novel phage receptor exists in 1.1954 that is absent in C600.

We investigated other mechanisms to explain why 1.1954 increases Stx production, focusing on those previously described or hypothesized given our knowledge of toxin regulation in O157:H7. One study (Iversen et al., 2015) found that 39% of commensal *E. coli* were lysogenized by the *stx2a*-converting phage φ734, from a highly virulent strain of STEC O103:H25. Of the 13 lysogens studied, 12 produced more phage when grown in the absence of inducing agents (mitomycin C or H_2_O_2_) than did the O103:H25 parent strain, suggesting that Stx2a levels would be higher in these strains as well. One lysogen, C600::φ734, was tested for Stx2a production and indeed this was found to be the case. Therefore, Iversen et al. (2015) proposed that lysogenization of commensal *E. coli* during an O157:H7 infection enhances overall toxin production. To the contrary, none of the five genetically distinct *stx2a*-converting phages tested in our study formed detectable lysogens in 1.1954. Lysogen formation can be inhibited if insertion sites are occupied by other phage, however analysis of the 1.1954 genome indicated most of the preferred insertion sites are unoccupied. Another study demonstrated that the DNase-colicins E8 and E9 can activate the SOS response, leading to greater toxin production when strains producing either are grown in co-culture with *E. coli* O157:H7 (Toshima et al., 2007). Our data also shows that microcin B17, which activates the SOS response through inhibition of DNA gyrase (Herrero and Moreno, 1986; Yorgey et al., 1994) does the same (Figure 7). Our data argues against the hypothesis that 1.1954 secretes DNA damaging molecules or other soluble factors known to regulate Stx2a production such as autoinducer 2 (Sperandio et al., 2001), and to the contrary suggests that physical contact between 1.1954 and PA2 is required (Figure 7). Although our bioinformatics analysis revealed that 1.1954 possesses a CDI system which may function as a tRNA anticodon nuclease, it may not function under our current laboratory condition. Future work should also consider whether other CDI systems previously described (type IV, V, and VI) may be involved (Aoki et al., 2005; Hood et al., 2010; MacIntyre et al., 2010; Souza et al., 2015).

Although SOS-mediated induction of Stx and phage production is the best understood pathway, several other mechanisms have been reported which serve as hypotheses for future experiments. For example, earlier reports revealed that lambdoid phage production is regulated through the capsular polysaccharide proteins RscA and DsrA (Rozanov et al., 1998), polynucleotide phosphorylase (Hu and Zhu, 2015), and RNA polyadenylation (Nowicki et al., 2015), and whether regulation of any of these are changed in O157:H7 upon co-culture with 1.1954 should be explored. Additionally, lambdoid phage production is under environmental control including salt concentration (Shkilnyj and Koudelka, 2007), pH and cationic chelators (Imamovic and Muniesa, 2012). The mechanisms behind these observations remain largely unexplored, however none of these were altered in our co-culture experiments and thus are not believed to contribute to differences in Stx production observed here.

In summary, our findings further define the *bamA*-dependent mechanism by which C600 increases Stx production. Although our study has not identified the exact mechanism for 1.1954, we provide evidence to indicate that a new mechanism exists which does not require BamA or act via secretion of a DNA-damaging molecule. This and other studies (Figler and Dudley, 2016; Matamouros et al., 2018) highlight the importance of appreciating strain-level diversity of *E. coli* when assessing how this organism affects health and disease outcomes.

## Author contributions

LX designed and performed all experiments, collected and analyzed data, and wrote the manuscript; HF contributed in manuscript writing; KG contributed in co-culture experiment; CH contributed in providing technical assistances; ED advised experimental design and manuscript writing.

### Conflict of interest statement

The authors declare that the research was conducted in the absence of any commercial or financial relationships that could be construed as a potential conflict of interest.
